# Does the design and implementation of proven innovations for delivering basic primary health care services in rural communities fit the urban setting: the case of Ghana’s Community-based Health Planning and Services (CHPS)

**DOI:** 10.1186/1478-4505-12-16

**Published:** 2014-04-01

**Authors:** Philip Baba Adongo, James F Phillips, Moses Aikins, Doris Afua Arhin, Margaret Schmitt, Adanna U Nwameme, Philip Teg-Nefaah Tabong, Fred N Binka

**Affiliations:** 1 School of Public Health, University of Ghana, P O Box LG 13, Legon, Accra, Ghana; 2 Mailman School of Public Health, Columbia University, 60 Haven Avenue, B-2, New York, NY 10032, USA; 3 Ghana Health Service, Ga East Municipal Health Directorate, P O Box AK 80, Accra, Ghana; 4 University of Health and Allied Sciences, P O Box PMB 31, Ho, Ghana

**Keywords:** Community-based health planning and service, Primary Health Care, CHPS Milestones, Urban Health, Health Policy, Ghana

## Abstract

**Background:**

Rapid urban population growth is of global concern as it is accompanied with several new health challenges. The urban poor who reside in informal settlements are more vulnerable to these health challenges. Lack of formal government public health facilities for the provision of health care is also a common phenomenon among communities inhabited by the urban poor. To help ameliorate this situation, an innovative urban primary health system was introduced in urban Ghana, based on the milestones model developed with the rural Community-Based Health Planning and Services (CHPS) system. This paper provides an overview of innovative experiences adapted while addressing these urban health issues, including the process of deriving constructive lessons needed to inform discourse on the design and implementation of the sustainable Community-Based Health Planning and Services (CHPS) model as a response to urban health challenges in Southern Ghana.

**Methods:**

This research was conducted during the six-month pilot of the urban CHPS programme in two selected areas acting as the intervention and control arms of the design. Daily routine data were collected based on milestones initially delineated for the rural CHPS model in the control communities whilst in the intervention communities, some modifications were made to the rural milestones.

**Results:**

The findings from the implementation activities revealed that many of the best practices derived from the rural CHPS experiment could not be transplanted to poor urban settlements due to the unique organizational structures and epidemiological characteristics found in the urban context. For example, constructing Community Health Compounds and residential facilities within zones, a central component to the rural CHPS strategy, proved inappropriate for the urban sector. Night and weekend home visit schedules were initiated to better accommodate urban residents and increase coverage. The breadth of the disease burden of the urban residents also requires a broader expertise and training of the CHOs.

**Conclusions:**

Access to improved urban health services remains a challenge. However, current policy guidelines for the implementation of a primary health model based on rural experiences and experimental design requires careful review and modifications to meet the needs of the urban settings.

## Background

The urban population in Africa is expected to double by 2030 with the fastest growing group being the urban poor [1,2]. Ghana exemplifies the concomitant problem associated with economic adversity and rapid urbanization. This growth lacks economic grounding, apart from the fact that migration is driven by growing rural poverty rather than expanding urban opportunities [3,4]. Over the years, rapid population growth in urban areas has not been complemented by equal economic opportunities and available public health services. Rather, it is often associated with deteriorating environmental conditions and a high demand for basic health and social services [5-9]. Providing health care access to the sprawling urban populations has become a major development issue, particularly in low-income countries lacking the financial and human resources necessary for the provision of health care services. Governments in most low-income countries are grappling with the challenges associated with providing high quality services and equitable access to health care to poor and underserved communities [10,11].

Since the late 1970s, the Government of Ghana has staunchly supported a policy aimed at extending the coverage of basic primary health care services to all citizens. The government, through the Ministry of Health, realized that this could be done by engaging the cooperation and authorization of the people at the community level. The policy therefore emphasized on the re-orientation of health workers to undertake community-based health care activities rather than the construction of huge hospital infrastructures [12]. In 2005, the Government of Ghana, the Ministry of Health, and the Ghana Health Service (GHS) adopted Community-Based Health Planning and Services (CHPS) as a national policy for the provision of primary health care services [13,14]. CHPS is a community-initiated health intervention, first piloted in Navrongo in the Upper East Region of Ghana. The CHPS initiative is a programme designed to translate innovations from the Navrongo experimental design into a national programme for improving accessibility, equity, efficiency, and quality health care services [15,16]. In this strategy, a trained nurse called a Community Health Officer (CHO) is re-oriented and deployed to live directly in the community they serve, providing health services to a catchment area called a zone. The CHO, with the assistance of trained community members known as health volunteers and a community health committee, render door-to-door health care to the community. This system of health service delivery has the ability to tremendously improve coverage of maternal, child health, and family planning services [17]. Evidence from the Navrongo experiment suggests that this strategy of delivering health services to underserved communities can tremendously influence maternal and child health indicators [18-20].

Although this policy was adopted nationwide, the process of implementing the strategy and the systems for delivering the services were based on rural experiences, as the initial experiment launching this paradigm shift in policy was introduced in the rural environment [15]. As a result, the implementation of CHPS has flourished significantly in the rural environment with little success found in urban areas [17]. The changing demographics of Ghana has manifested in a rapid population growth in urban areas. For example, Ghana’s population has increased from about 8 million in the 1960’s to 24 million in 2010, with an urban growth rate of 3.9% as against a rural growth rate of 1.0%, indicating that over 51% of the population now reside in an urban area [21]. With this level of urban growth, which is mainly fuelled by the continuous rural–urban migration, most of the rural health problems are being transferred to the cities. The fact that these populations are relocating to urban areas requires that adequate health care services are provided to meet their new health needs. CHPS could be a key strategy to provide basic primary health care services to populations living in poor underserved urban communities. Some scholars are of the view that best practices can be derived from an analysis of existing practices than trying to devise best practices from first principles [22]. In order to address all of the considerations required for making CHPS function for the urban poor, it is important to examine which aspects from the rural model will succeed and which are deemed inappropriate. In recognition of this, CHPS was introduced to the Ga East municipality of the Greater Accra region, with the primary aim of testing the suitability of the rural strategies in an urban setting. This paper seeks to identify and document the various implementation challenges and lessons derived from introducing the rural experiences into an urban environment, including proposing alternatives to these strategies based on practical experiences aimed at enhancing the appropriateness of such tactics for the urban environment.

## Methods

### Study area

This study forms part of a broader quasi-experiment designed to test proven health innovations in Ghana (Ghana Essential Health Intervention programme, GEHIP) and Tanzania (Tanzania Essential Health Intervention Programme, TEHIP) on maternal and child health [23]. The study was carried out in the Ga East municipality of the Greater Accra region, which has a large concentration of informal settlements serving as a residence for many rural to urban migrants. The municipal capital is at Abokobi and covers a total land size of about 166 km^2^. The municipality is divided into four zones with 16 operational areas consisting of 42 communities. The 2010 Ghana’s Population and Housing Census indicated that the total population of the municipality was 259,668, with 127,258 and 132,025 representing the male and female populations, respectively [21]. The municipality is divided into four sub-municipalities for the organization of primary health care services, namely Madina, Danfa, Taifa, and Dome.

### Ethics statement

Ethical approval for this study was received from the Ethics and Institutional Review Committee of the GHS and the Navrongo Health Research Centre in the Upper East region of Ghana. The Greater Accra regional branch of the GHS was subsequently informed of the intervention and institution approval received from the GHS. A baseline study was first carried out to provide data that could be used to monitor the progress of the intervention. Written informed consent was sought from all respondents which were mainly women in their reproductive ages (15 to 49 years old). Participants were further informed of their right to withdraw from the study at any time without any punitive measure taken against them. Personal identifiers were not taken and, if accidentally taken, they were removed from the data before analysis.

### The urban pilot study

Following the baseline survey, the pilot phase of the urban CHPS was then launched in the intervention sub-district (Dome). The urban CHPS initiative is designed to test whether the approaches of ‘bringing services to the people’, and strengthening communities to ‘bring health to themselves’, can overcome barriers to reproductive health and improve child survival in urban and peri-urban slum areas, thereby promoting health equity within urban neighbourhoods. It aims to improve access to quality health care, expand the referrals of cases/case referrals, and to increase the health knowledge of the community. After a series of consultations and meetings with key stakeholders, including members of the community, political authority and the health sector, a formative qualitative appraisal was conducted to determine the form and nature in which urban CHPS could be implemented. The qualitative formative research provided information on the social composition of the urban population, health-seeking behaviours for infant, child, and maternal health, health decision making at the local level, social factors influencing healthcare provision, the community conceptualization of Urban CHPS, and potential models for the programme’s design.

Upon the completion of the qualitative appraisal, two models of urban CHPS, one using the rural milestones and a modified version were developed and tested in the Dome sub-district of the Ga East District. Two zones, Ayigbe and Grushie, were created in separate communities for the pre-testing. The rural milestones were tested at Ayigbe town whilst modifications to the rural milestones were tested at Grushi town. The pilot study was conducted from September to December 2011 focusing purely on maternal and child health-related interventions. Several lessons were learnt from the two models, which led to some modifications and subsequent scale up.

### Data collection and analysis

The routine data collected were reviewed monthly and analysed to inform the adoption of the new strategies. The coverage and other daily reports from the CHOs was compared. Simulations were employed to test which strategies would be appropriate for the urban settings. A number of strategies were adopted and tested until a particular strategy emerged as an appropriate one in the intervention communities. Additionally, another qualitative appraisal was carried out to elicit information from stakeholders on the most appropriate strategy for the urban setting. The operational differences between the rural CHPS and the urban CHPS were then documented and have been presented in the results in the form of narratives.

## Results

### Comparison between rural and urban CHPS milestones

There are six milestones involved with the implementation of CHPS. The first milestone is the process of community-based planning. This involves the mapping and zoning of the community and the creation of community health compounds (CHC). Community entry is then carried out to enable for participatory implementation of the intervention. To be able to accommodate the CHOs within their zones, the community usually constructs a structure that serves as a CHC (clinic) and as a residence for the CHO. However, where an existing structure is available, it is often refurbished for this purpose. As a measure to facilitate the activities of the health care workers, essential equipment is procured as part of the milestones in CHPS implementation. This milestone is followed by the orientation of the nurses, training them with the knowledge and skills that are relevant for effective community engagement and service delivery. Since CHPS is a community-based initiative, it is important to develop and maintain an effective link between the CHOs and the community. Therefore, health volunteers are often selected by the community, trained, and deployed to assist the work of the CHOs.

### Community-based planning

The first milestone in the CHPS strategy is carrying out a situational analysis of the area. Chiefs, who are the custodians of the land and have responsibility over the residents in rural areas, play a very significant role in assessing the existing situation that will serve as a baseline for further consultations and subsequent implementation of the programme. Starting the CHPS process involves delineating zone boundaries and assessing work force requirements and capacities. Defining the geographical community to constitute a zone in rural areas often reflects the typical rural settlement patterns, which conforms to extended family systems. Typically, such settlements are often under the authority of a common head. This milestone is different in urban areas because of the settlement pattern and the lack of centralized traditional authority to which all community members’ respect. Contrary to the homogenous nature of the rural dwellers, the urban sector is an amalgamation of people from different ethnic backgrounds. Demarcating the zones in the urban environment requires creating blocks and identifying neighbourhoods that may conform to ethnic groups. It also involves defining geographical responsibility within multi-ethnic groups.

### Community entry

In Ghana, community entry and sensitization are strategies which are adopted to stimulate dialogue and encourage communal support and cooperation for health programmes. Community entry involves developing leadership and initial participation in the programme through discussions with community leaders and residents. In a rural setting, this milestone is achieved through building an understanding with chiefs, elders, and opinion leaders in the community. The chiefs and elders will then organise durbars, which are traditional gatherings that typically include drumming and dancing with the elders and chiefs, and can be used as a platform to communicate the impending intervention as well as to solicit the support of community members. Committees can also be formed during such community gatherings to facilitate the implementation of the intervention. Again, this strategy was found less appropriate in the urban environment. To be able to achieve this objective in the urban areas, focus should be directed at identifying social network groups, which may also correspond to ethnic settlements and other mutual community organisations. In the absence of traditional leaders, formal authorities and politicians, such as assemblymen, District and Municipal Chief Executives, and the Members of Parliament, should be engaged to serve the role that traditional leaders play in the rural context. Durbars can also be organised through the facilitation of such network groups and formal political authorities to sensitize the community members on the intervention.

### Facility development

This milestone on CHPS implementation involves building a CHC that will serve as the clinic for the provision of health care services. Community health services require a simple facility that provides a room for the CHOs to reside in and a separate space to serve as the clinic. Developing such facilities contributes to the sense of ownership of the project by the community. It also creates a platform for involving community leaders in planning and resource mobilization. However, in the urban areas the non-availability of land that could be donated for the construction of the CHC creates the need for a modification of the rural strategy. Rooms therefore had to be rented for CHOs and temporary mobile structures constructed to serve as clinics. In some instances, CHOs live outside their zones and commute daily to their assigned areas in order to deliver health services.

### Procurement of essential equipment

Provision of essential equipment is an important milestone for CHPS implementation. The equipment facilitates the work of the CHOs as well as the community health volunteers. For rural CHPS, CHOs/community health volunteers are provided with motorbikes and bicycles to facilitate their movement around their catchment areas. In addition, the CHOs are provided with the equipment required to render maternal and child health care interventions. However, motorbike and bicycles are unsuitable in urban areas, with low-cost three or four-wheel vehicles deemed more appropriate. In addition to the maternal and child health related equipment procured for rural implementation activities, the urban health strategy had to procure additional adult health-related equipment. For example, equipment for monitoring blood sugar levels and blood pressure has been introduced due to the emergence of non-communicable diseases among the urban poor. Considerations for the provision of adolescent friendly health care service are also being prioritized, considering that adolescents dominate urban population demographics.

### Nurse orientation to community work and posting to community health compounds (CHCs)

The most critical stage of the CHPS process is the launching of the CHO into the community. This step is accompanied by an introductory durbar celebrating the onset of the door-to-door health care services to be provided to the community. The CHO services include the provision of clinical care at the CHC, conducting household visits to provide family planning services, health education, and ambulatory care, and implementing outreach clinics for childhood immunization. In addition to these activities carried out by CHOs in the rural CHPS, urban CHOs also require an urban-focused orientation on health education, which integrates adolescent- and adult-oriented health services. The lack of traditional social structures, formal organizations and the relatively disorganized settlement patterns among the urban poor communities also make them vulnerable to disasters. Therefore, orientations on disaster management are needed for CHOs in urban areas.

### Volunteer identification, recruitment, training, and deployment

Central to the rural CHPS model is the role of community health volunteers and committees who are often selected by community members and trained to assist the CHOs in providing health care to people within their zones. Community members’ usually select community volunteers to be trained by the GHS for a six-week period prior to deployment back into their communities. Volunteers serve as health activists within their community, creating awareness on health issues and its social determinants and mobilizing the community towards local health planning and increased utilization of existing health services. The training of volunteers is usually a combination of classroom teaching and field attachments, covering topics such as basic first aid, family planning, and health promotion strategies.

Although the training of volunteers in the rural and urban areas is similar, their activities differ significantly. Whereas rural volunteers engage in basic medicinal distribution and health promotion including distribution of bed nets, condoms, and oral rehydration solution, urban volunteers do not engage in such activities and remain less focused on disease-specific interventions. Rural volunteers are also always indigenous to their community and therefore are found to be more willing to offer their services for no compensation, whereas their urban counterparts were unwilling to offer their time for free. The presence of periodic or seasonal disease-specific health interventions in rural areas also creates the opportunity for volunteers in the rural areas to get periodic stipends, which serves as motivation. Within the urban context, it was also found that the use of community health committees was more effective than the use of volunteers. Table 1 provides a summary of the varying rural and urban considerations when adopting the CHPS milestone model for implementation.

**Table 1:** Rural and urban considerations for CHPS milestone development

**Milestones for the development of CHPS**	**Rural considerations**	**Urban considerations**
Community-based planning	Situation analysis, initial outreach to chiefs, ‘zoning’ of catchment areas	Block and neighbourhood identification, clarification of geographic responsibility
Community entry	Building understanding with chiefs, elders, and opinion leaders	Focus on identifying social networks (corresponding to ethnicity of settlers)
	Developing community health	Outreach to formal authorities and politicians
	Organizing CHC action	
	Developing durbars for health communication	
Essential equipment	Motorbikes and bicycles in addition to clinical equipment for Integrated Management for Childhood Immunization (IMCI), Expanded Programme for Immunization (EPI), Family Planning/Reproductive Health (FP/RH)	Low cost 3 or 4 wheel vehicles, in addition to clinical equipment for IMCI, EPI, FP/RH. Adolescent and adult health important
Facility development	Community volunteer construction of CHC or renovation of existing facility	Arranging donation of secure space or renovation of donated space; no CHC
Nurse community engagement training and posting	Training in community entry, liaison, and sustaining community participation	Training in health education in the urban context
Volunteer identification, training, and deployment	Community organizational focused; basic medicinal distribution; health promotion including bed nets, condoms, oral rehydration solution	Service focused volunteers with no curative services (more limited role)

### Implementation and lessons learnt during the pilot study

The lessons learnt over the duration of the six-month pilot introducing the CHPS model to an urban setting proved relevant in guiding discourse focused on adapting strategies to meet contextual needs. These CHPS policy modifications for the urban environment are summarized in Table 2.

**Table 2:** Key lessons learned from ‘Phase 1’: the urban pilot and research activities

**WHO pillar/specific activity**	**Rural CHPS strategy**	**Urban CHPS findings**
**The provision of essential health services and technologies**		
The burden of disease	The rural burden of disease is dominated by three causes of childhood morbidity and mortality: malaria, acute respiratory infections, and diarrheal diseases.	The disease burden is much more diverse in urban locations. Although the disease transition is underway, a large part of the population remains impoverished and susceptible to infectious diseases. Considerably, urban health planning must cater for infectious-, chronic-, trauma-, and injury-related morbidity. Furthermore, the high concentration of adolescents in urban areas means that sexually transmitted infections are of serious concern.
The role of health campaigns	In rural settings, the CHPS program frequently utilizes disease-specific health campaigns, such as mass immunization days or entire cadre focused on particular modalities or syndromes of illness.	Need to consider the diverse burden of disease in the urban context, as well as the lack of seasonality – the vertical campaign-based system is not relevant.
The provision of ‘doorstep’ care	Doorstep services are needed for family planning service effectiveness, but less needed for the provision of ambulatory child health service.	The ‘doorstep model’, which is central to rural CHPS operations, proved inappropriate within the urban context. Much of the population are in full-time employment during the day and were not present for these home visits. To accommodate for this, the CHPS nurses began providing evening and weekend home visiting schedules.
		Use of specialised outreach services, visiting available for mutual groups on designated days.
The provision of family planning and reproductive health services	Unmet need for spacing comprises nearly all of the demand for services.	The urban population had more interest in using family planning for limiting rather than childbearing as typically observed in rural populations.
Extending service access	Private facilities and services are less important than the Ghana Health Service public sector program.	The urban populations were more likely to utilize private sources, such as pharmacy shops and other drug vendors when seeking for health resources. The public sector plays a much less prominent role than in rural communities.
**Manpower, training, and deployment**		
Training of nurses	Training should be focused on the Integrated Management of Childhood Illness and family planning. Midwifery training is important as result of greater likelihood of home-based delivery.	Urban nurses should focus more on adolescent and adult health needs, in addition to chronic diseases and sanitation. There should also be a greater emphasis on in-service trainings, due to the breadth of competencies required in working with this disease-diverse population. Midwifery is also of less importance for urban nurses due to the greater likelihood for facility-based deliveries.
Volunteer involvement	Appropriate engagement of traditional leaders can facilitate sustainable volunteerism.	Engagement and support of volunteers remains at the core of the CHPS model. Urban volunteers, however, indicated that compensation was necessary for their participation, due to the greater opportunity costs for their involvement, thus challenging the traditional CHPS volunteer paradigm.
	Involving men in family planning is crucial to success; volunteers can play an essential role in developing male mobilization and participation in family planning.	Individual men are more accepting of family planning in urban areas, obviating the need for group outreach; volunteer outreach to male social networks does not work in the urban context.
**Information for decision-making**		
Mobile-Health (m-health) for health service support	Cell phone connectivity is a problem in rural areas; cell phone information services to rural women often fails to connect with intended recipients.	Cell phones are ubiquitous and functional; gender problems that hamper access in rural areas are less relevant to the urban context.
**Essential supplies and logistics**		
Resolve access problems by solving logistical challenges	Motorbikes and bicycles are required for supporting ‘doorstep’ services.	The established CHPS model requires nurses to remain resident at the CHPS health post. This precedent, however, proved difficult due to the more expensive nature of urban real estate. The provision of accommodation for nurses was costly, and financial constraints required for the rental rather than purchase of health post property.
**Essential resources and planning**		
Health insurance	Trust arrangements permit workers to provide services with the understanding that extended families will eventually reimburse providers for commodity costs.	Traditional ‘trust as insurance’ arrangements do not work in the urban context. There is much higher coverage of the national health insurance programme in urban areas. These populations therefore seek health services directly from public facilities. Home-based (‘doorstep’) care is not always supported by health insurance, which questions its appropriateness for this population.
Organizational capacity	Rural setting offer opportunity for stable population.	The urban setting offered increased opportunities to mobilize populations, including through youth groups, women’s market associations, beauticians, unions, political entities, and church organizations.
**Leadership and governance**		
The role of Health Committees	Existing mechanisms for social organization, networks, and lineal structures can be marshalled for organizing the governance of primary health care operations.	Health committees (HCs), which include community representatives from the designated CHPS zone, are central to CHPS development and operations. In the urban context, HCs lack the influences of traditional social structures, including tribal leadership. In the absence of such arrangements, local politics plays a much greater role in the formation of HCs. In addition, the HCs sought for greater authority and for the provision of a permanent location to operate from. For example, the HCs sought the power to be able to give out sanctions to individuals that violated established sanitation standards within their communities.
Developing community support	Communities will organize leadership for CHPS and construction of health posts.	Although there was extensive support for the introduction of the CHPS project in pilot communities, actual participation was sporadic and disorganized. This was likely due to the lack of traditional chieftaincy and lineage systems and ethnic diversity, resulting in challenges in mobilizing collective action.
The social context	Existing social institutions can be used for organizing services and simplifying outreach. Women will commit time to activities that involve existing social groups.	Opportunity costs are high for urban women and every family member must contribute financially. This results in serious time constraints for urban women. Childcare needs are great, especially in the absence of extended family networks. Furthermore, this lack of familial support results in less social protection, including child protection and social insurance, in addition to increased social discord and crime.
Adapting strategy to the gender context	Rural women are often dependent on males for key decisions and economically.	Urban households are often more dependent on women. Women often hold an economic role within their families, and often times heading their households. Marital dissolution is more common in urban locations.

## Discussion

### Urban CHPS: future design and implementation considerations

Since CHPS activities in the rural settings had positive effects on maternal and child health [18-20], there have been several efforts aimed at expanding the implementation of CHPS in both rural and urban settings across Ghana [14]. This study was therefore conducted to determine both the common characteristics and differences between the rural and urban health interventions, based on the milestones framework of CHPS, including providing practical suggestions on ways to effectively adapt CHPS to better serve urban population needs. The results of the study indicate that the lack of organisational structure and centralised traditional leadership in urban communities is a major challenge to the introduction of the urban CHPS. Considering that, a key principle of CHPS introduction involves the traditional leadership of a community accepting the CHPS concept and committing themselves towards supporting it. CHPS relies on this participation and mobilization of the traditional community structures for service delivery [24,25]. Community participation is vital, as it is a strategy that can be used to marshal the resources available in a community towards activities relevant to the sustainability of the programme. For example, in rural areas, the community can provide labour and land for the construction of clinics and residential facilities for CHOs. Community investment in turn generates sustained community interest and involvement in the programme [26-28]. To be able to successfully implement urban CHPS, the support of organized mutual groups and political heads remains pivotal. These groups are capable of filling in the gap that is created in the urban community by the absence of traditional leadership.

Countries reforming their health sector appreciate the importance of involving community members in health service assessments and priority setting through decentralisation policies [29,30]. This is a very important consideration in both rural and urban CHPS. Although urban communities are densely populated with increased human resources, their relative ethnic diversity undermines the sense of collectiveness, which is an important aspect of community participation. To be able to address this challenge in the implementation of urban CHPS, the programme should reach out to existing social networks and established groups within the community. To help sustain the interest of urban populations, leaders from religious organisations as well as educational facilities should be actively engaged in the activities of the intervention. Private health institutions and chemical shops that operate within the catchment areas should also be viewed as primary stakeholders and collaborations should be established between these institutions and the CHPS initiative. Frequent community meetings are also required to provide a platform for discussion on ways to improve upon the community-based health care services.

The availability of public health facilities in rural and urban areas also creates a distinction between these CHPS strategies. In rural areas, public health facilities are often unavailable whereas urban settlements are often surrounded by a plethora of private health facilities. Such venues are generally patronised by the elite, however, as opposed to those living in the surrounding impoverished communities. The concept of health for the urban poor is therefore very different from the rural poor. The urban poor generally view health and the use of health facilities as a privilege for the elite, having been neglected from economic access to such facilities for several years. It is therefore imperative to note that some members of the community will consider any attempt to make health services accessible to them through CHPS as a way of increasing access only for these elite groups. Slum dwellers, who have often been denied access to civic engagement, have no effective means to protect themselves and demand for services to be provided and for accountability to exist for those who provide them [31,32]. Identifying and engaging the leadership of various ethnic groups is important for the cooperation of community members.

The concept of volunteerism is also different within urban communities. Community health volunteers have been used in a number of programmes to encourage community involvement and to compensate for the shortages of health professionals in the health sector [25]. Unlike volunteers who work in rural CHPS who are often willing to offer their services free, volunteers in urban areas demand incentives due to the higher opportunity costs in providing 3 to 4 hours of their time a day for voluntary work. Failing to motivate volunteers could therefore endanger the programme because of the possibility of high attrition. Retention of village health volunteers in health intervention programmes greatly influences their sustainability, a high attrition rate among health volunteers may jeopardize the maintenance of programme activities [33]. The impact of incentives has been found to positively influence the efficiency of public health interventions in Ghana [34]. Nevertheless, remunerating community health volunteers raises questions about sustainability and health financing. Previous studies have reported that community volunteerism is short-lived since volunteers usually expect compensation for their work, but in most instances the communities they work for are very poor and unable to afford these incentives [35]. Within this context, more emphasis should remain on the creation of community health committees whilst continuing to explore ways to compensate volunteers. Governments in developing countries should consider engaging the services of retired workers who are still physically active as health advocates and community health volunteers. In many communities, these retirees are highly respected and their involvement in health advocacy may yield positive results. However, such notions require further assessment on their feasibility in practice.

The ‘doorstep model’, which is central to rural CHPS operations, proved inappropriate within the urban context. The doorstep model requires CHOs to be resident in the community; however, urban communities cannot provide accommodation for the CHOs within the zones, as that requires the costly rental of rooms for CHOs. In situations where rent cannot be acquired within the zones, the CHOs will have to live outside the zone and commute daily to the zone to provide health services. This can be enhanced with mobile technology for health since the telecommunication networks in urban areas are highly effective and gender barriers in the use of mobile telephones do not exist as in rural areas [36,37]. A good link between the numerous private health care providers and the CHOs working within the community can also further facilitate referrals during times that the CHOs are unavailable to serve the community. The urban areas are also devoid of many of the transportation challenges found in rural areas in facilitating referral systems.

Outreach services also differ significantly between rural and urban settings. In the urban context, service outreach should also be targeted at organised groups and community gatherings such as market places, churches, and mosques. Trust arrangements in the rural areas permit health workers to provide services with the conviction that extended families will eventually reimburse providers for commodity and service costs. However, traditional ‘trust as insurance’ arrangements do not work in the urban context. Furthermore, there is much higher coverage of the national health insurance programme in urban areas. Considerably, the urban populations are more likely to seek health services directly from the available public facilities. Home-based (doorstep) care is not always supported by health insurance in urban areas. Another area of variance involves the routine home visits practiced by rural CHOs, where services are generally delivered in the mornings and afternoons. This time, the home visiting schedule was found unsuitable in the urban areas. Many homes in urban areas are empty during these hours due to more of the population participating in the workforce. Therefore, night and weekend home visiting schedules had to be introduced to ensure better coverage.

Rural and urban poor families differ significantly in terms of headship and decision-making. Whereas a typical rural family is patriarchal, with men serving as the heads and main decision makers, urban families in informal settlements are more dominated by women. These differences reflect the life style of rural/urban culture and concepts of family planning. Family planning services is fundamental in the rural CHPS as a strategy to reduce fertility. Involving men in family planning is crucial to success as men usually make critical family decisions including those related to reproductive health in rural areas. Volunteers can play an essential role in rural areas by developing male mobilization strategies towards participation in family planning. However, individual men in urban areas are more accepting of family planning obviating the need for group outreach. Hence, volunteer outreach to male social networks does not prove as effective in the urban context.

The burden of disease also differs considerably within the rural and urban context. The rural disease burden for children is dominated by malaria, acute respiratory tract infections, and diarrheal diseases, with high prevalence during the rainy season. In contrast, the overlap between the rainy and dry season in urban areas make these conditions prevalent throughout the year. Therefore, disease-specific and seasonality based approaches found in rural areas are inappropriate in the urban context. Prevention of sexually transmitted infections and adolescent health are two key areas of concern in urban areas because of widespread commercial sex practices. As noted, environmental hazards are more widespread in urban areas, with children more prone to experience trauma, injuries, and endangerment from natural disasters. The demographic characteristics of the urban residents are characteristically different from the rural settings. The urban population is made up of mainly adolescents, re-enforcing the need for access to adolescent friendly health services in urban settings. It has been estimated that as many as 60% of all urban dwellers will be under the age of eighteen by the year 2030 [38].

The training of rural nurses during their re-orientation exercises are generally focused on the integrated management of childhood illness and family planning in rural areas. Working knowledge on midwifery is also required because of the sometimes non-availability of health facilities in rural areas and the high prevalence of home deliveries. However, urban nurses should focus more on adolescent and adult health needs, chronic diseases, and sanitation. There should also be a greater emphasis on in-service training due to the breadth of competencies required in working with this disease diverse population. Midwifery is also of less importance for urban nurses due to the greater likelihood for facility-based deliveries.

## Conclusions

Although the current CHPS model proved effective in promoting rural health, this model cannot be transferred directly to urban settings due to many contextual differences that exist in these environments. The policy must therefore endure a careful review process aimed at catering for and adopting the model based on the complexities of the urban environment. This should involve acquiring from the rural strategies all that is suitable for the urban setting and also considering the need for modifications for urban-specific issues. Based on these findings, a new urban-focused policy should be developed aiming at better addressing the challenges identified that will allow the effective utilization of resources inherent in the urban environment for the implementation of CHPS.

## Abbreviations

CH: Health Committee; CHC: Community Health Compound; CHO: Community Health Officer; CHPS: Community-Based Health Planning and Services; GHS: Ghana Health Service.

## Competing interests

The authors declare that they have no competing interests.

## Authors’ contributions

PBA, JFP, MA, DAA, MA, MS, and AUN conceived the design; PTNT and FNB took part in data collection and analysis; PBA, JFP, MS, MA, PTNT, and AUN wrote the manuscript. All authors read and approved the final manuscript.
